# “Finding happiness in daily work”: an ecological study on the emotions of novice EFL teachers in rural primary schools in China

**DOI:** 10.3389/fpsyg.2023.1275045

**Published:** 2023-11-30

**Authors:** Yujing Yao, Jie Xu, Conggen Yan

**Affiliations:** Chinese Education Modernization Research Institute, Hangzhou Normal University, Hangzhou, China

**Keywords:** teacher emotion, Bronfenbrenner’s ecological system, rural teacher, qualitative study, teachers’ professional development

## Abstract

While research on teacher emotions has grown in the past decades, little is known about rural teachers’ and novice teachers’ emotions. Based on ecological theory, this study selected two novice EFL teachers as the research objects. The goal was to explore their emotional experiences and the factors that influenced them over 3 years while teaching in rural primary schools. The research data was collected primarily through semi-structured interviews, teaching diaries, and narrative frameworks. Three-step coding and topic analysis were used to analyze the collected data. The data analysis revealed that the two novice teachers generated 62 emotions while interacting with various ecosystems. In Microsystems, teacher-student interaction has a strong influence on participants’ emotions. Two participants experienced 19 positive emotions and 9 negative emotions during their interactions with the students. In addition, novice teachers may experience negative emotions if they are burdened with too many non-teaching tasks. In this study, two participants were able to effectively manage their negative emotions. The general emotional trend was positive, which motivated them to stay and continue teaching in the rural area. The results of this study have implications for the professional development of rural teachers and novice teachers.

## 1 Introduction

Teaching is an emotional practice (Hargreaves, 2000), and Schutz and Lanehart (2002) argue that “emotions are intimately involved in almost every aspect of the teaching and learning process” (p. 67). In the past, the topic of teacher emotion has received much attention from psychologists and educational scholars. Many studies have revealed to us the importance of teachers’ emotions. From a teacher’s perspective, emotions play a role in the development of teachers’ professional identity and well-being (Day and Qing, 2009; Fried et al., 2015). Teachers’ emotions also affect their performance at work and their productivity (Day, 2011). Positive emotions can support teachers to generate innovative strategies or methods to solve problems and improve teaching effectiveness (Chen, 2019). For example, as a positive emotion, studies have found that primary school mathematics teachers’ enjoyment of teaching is positively correlated with their teaching time and attitude toward student struggle. Teachers with positive emotions toward teaching tend to spend more time teaching and have a positive attitude toward student struggle (Russo et al., 2020). In a study conducted in the field of higher education in Australia, there was a relationship between the emotional experience of university teachers and the teaching style, and teachers with positive emotions tended to adopt student-centered teaching methods (Trigwell, 2011). A study of primary school teachers in Hong Kong, China, also obtained similar results (Chen, 2018a,b). However, excessive negative emotions may impair teachers’ creativity and flexibility (Becker et al., 2014), weaken their ability to build multiple psychological resources (such as resilience) and reduce their happiness (Zheng et al., 2022). It will affect job satisfaction (Atmaca et al., 2020) and even lead to teacher burnout or turnover (Akin et al., 2013; Burić et al., 2019; Torres, 2020). From the student’s perspective, the emotional transmission between teachers and students is two-way (Frenzel et al., 2018), and students’ emotions are influenced by teachers’ emotions (van Uden et al., 2014). As learners in the classroom, they have more contact and interaction with teachers than other people (such as parents), so students’ class participation, learning motivation, and their relationship with teachers are all related to teacher emotion (Yan et al., 2011; Becker et al., 2014). When students perceive that teachers are bored, their learning motivation will decrease (Tam et al., 2019). In addition, teachers troubled by negative emotions may be less caring and tolerant of students and unable to maintain an excellent teacher-student relationship. Frenzel et al. (2020) pointed out the negative correlation between anger, anxiety, and the assessment of the teacher-student relationship. There is also some evidence that teachers’ emotions correlate with students’ academic achievement over time (Klusmann et al., 2016; Frenzel et al., 2021). Emotions not only affect the professional development of teachers (Mansfield et al., 2012) but also play a role in the growth of students.

There is an increasing discussion around teacher emotions, and some scholars tend to study teacher emotions quantitatively, e.g., (Chen, 2016; Lavy and Eshet, 2018). There is no denying that this approach works. However, because emotion is implicit, more than simple quantitative research is required to fully and deeply understand teacher emotion. Some scholars study teachers’ emotions from the ecology perspective and systematically explore teachers’ emotional experiences based on specific events (Gu and Gu, 2015). This qualitative method provides new ideas for the study of teachers’ emotions. Nevertheless, in general, most of the current research on teacher emotion comes from the West (Uitto et al., 2015; Atmaca et al., 2020), and the study group is mostly teachers of schools in developed cities. There is limited research on teachers’ emotions in the Asian context, especially in the rural context.

In addition, novice teachers face many challenges at the beginning of their work as they transition from learning in regular colleges to teaching in a natural classroom environment, and they also need to face the emotional needs of colleagues, students, and parents (Lee and Yin, 2010; Cross and Hong, 2012). It is, therefore, understandable that some people view the first years of teaching as a highly complex process involving a wide range of emotional dynamics (Mehdizadeh et al., 2023). However, most current studies on novice teachers focus on professional quality development and ability improvement, and only some people carry out longitudinal studies on novice teachers’ emotions (Nazari et al., 2023).

Driven by these gaps, this study will examine the emotional development of novice EFL teachers at the beginning of their careers. In short, we longitudinally tracked the emotional experiences of two novice EFL teachers at the beginning of their work, aiming to understand the emotional development of novice EFL teachers during their interaction with different environments. Our research questions are as follows: (1) What is the emotional development of rural novice EFL teachers at the beginning of their work? (2) What factors affect the emotions of novice EFL teachers in rural areas? Based on teachers’ personal experiences, this study explores teachers’ emotional flow from an ecological perspective, showing the complexity and dynamics of teachers’ emotions. Many data about the emotional experience of rural novice teachers is provided, which is helpful to enrich the research content of teachers’ emotions. Our research also provides insights into the retention and development of novice teachers in rural areas.

## 2 Literature review

### 2.1 Emotions and teacher emotions

In 1996, “teacher emotion” appeared in a special issue of the Cambridge Journal of Education on emotions. Subsequently, researches on “teacher emotion” gradually increased and became an important topic in the current education field. Emotion is a complex concept. By nature, a person’s emotions are dynamic and because the definition of emotions involves multiple subject areas (not only education and psychology), scholars have defined emotions differently. If combining physiology and psychology, we can explain emotions as the changes and experiences that individuals undergo both physically and psychologically. According to Hall and Goetz (2013, p. 5), emotion is “a multidimensional structure consisting of emotional, psychological, cognitive, expressive, and motivational components.” Emotions in education are constructed in society (Schutz et al., 2006), so some scholars have incorporated sociological perspectives to explain teacher emotions. In this paper, we use the definition from Schutz et al. (2006). He explains emotions as “socially constructed, individually formulated ways of being that arise from conscious or unconscious judgments about perceived success in achieving goals or maintaining standards or beliefs in transactions that are part of the socio-historical context” (p. 344). Clearly, what distinguishes this view from the definitions given by other scholars is that it takes into account the influence of external environmental factors on teacher emotions and investigate teacher emotions with a developmental, integrative perspective.

There are various approaches to classify teacher emotions. The most common one is to classify teacher emotions into positive and negative categories. For example, joy, excitement, and pride are positive emotions, while negative emotions include anxiety, anger, and frustration (Hargreaves, 1998; Taxer et al., 2019). The dichotomy laid the foundation for other subsequent methods of classifying emotions, but some scholars have argued that it is too simplistic to categorize teacher emotions as positive and negative (Kristjánsson, 2016). And later, Parrott (2001) organized teacher emotions into a multilevel structure for classification and enumerated multiple emotions. That is, each basic emotion contains secondary emotions, which also contains a third emotion. For example, the secondary emotions of love are desire, pleasure, etc. This study will refer to this classification and finally classify the participants’ emotions into positive and negative categories.

It is worth mentioning that some scholars have developed models of teacher emotion, which provide more ideas for in-depth research on this topic. Frenzel (2014) proposed a reciprocity model of teacher emotion, which clarified the relationship between teacher emotion and student achievement, misbehavior, teacher-student relationship, and teaching effect. Fried et al. (2015) reviewed the literature on teacher emotion published from 2003 to 2013 and developed a conceptual model of teacher emotion. The model consists of four concentric rings, covering the influence factors, functions, and four kinds of complexity of the teacher emotion concept. Among them, teachers’ personal characteristics, evaluation, and social culture influence teachers’ emotions. Informing, motivating, regulating, influencing cognition, and giving experience quality are the five functions of teachers’ emotions. This model provides enlightenment for the subsequent teacher emotion research, but some scholars think it lacks teachers’ characteristics. Based on this, Chen (2020) constructs a new teacher emotion model by collating more than 800 relevant articles. The model is composed of anthems, mediators, and consequences. Teachers’ personal, situational, and emotional abilities are the anthems of teachers’ emotions. The definition, category, and measurement of teachers’ emotions are the mediators, while consequences are divided into four aspects: teachers, students, teaching, and learning. Compared with previous models, Chen’s model is more detailed and comprehensive, focusing on the interrelationship between teachers’ emotions and various aspects.

### 2.2 Research on teacher emotion from an ecological perspective

The continuous deepening of theoretical and practical research makes people realize that teacher emotions are related to their surroundings and interactions with others (Schutz et al., 2006). Teacher emotions are not generated within the individual teacher, but are feelings that arise as a result of the teacher’s interaction with others in a given environment (Farouk, 2012). Scholars have then studied teacher emotion from an ecological perspective, which is particularly common in studies of language teaching (Chu et al., 2021). Cross and Hong (2012), for example, conducted a case study on two elementary school teachers. The two elementary school teachers work in a special school where they deal with students from high-poverty families and diverse ethnic minorities. Surprisingly, in this adverse environment, both of them, despite some unpleasant experiences, still demonstrated high levels of satisfaction and enthusiasm, and showed more positive emotions. This study provides elicitation for subsequent research, and a growing number of scholars are drawing on Bronfenbrenner’s ecosystem theory for teacher emotion research. Chen (2017) interviewed 53 teachers from Hong Kong and mainland China, and the study reported 68 kinds of teacher emotions (half positive and half negative). In the study, the teachers experienced up to 50 emotions in the microsystem, but only 10 emotions in the macrosystem, which was farther away from them. In other words, in an ecosystem with five nested settings, the closer the teacher is to his environment, the more emotions he experiences, and vice versa. Up to half of the negative emotions aroused the researcher’s curiosity. So in 2019, she surveyed more than a thousand elementary school teachers in China using a mixed-methods study in an attempt to understand the underlying factors influencing their emotions. Through the content of interviews with 25 teachers and questionnaires with more than 1,000 participants, the results revealed that participating teachers experienced high intensity emotions (positive and negative) at the microsystem level. Some factors from the macro level (e.g., excessive and unrealistic expectations of students’ parents, harsh public accusations) bring negative emotions and unavoidable stress on teachers (Chen, 2020). There are more and more international researches on teacher emotion, with some scholars beginning to focus on a certain aspect of teacher emotions and others turning their attention to subsidiary teachers. Hofstadler et al. (2020) conducted semi-structured interviews with 16 Austrian CLIL teachers and used ecosystem theory to understand how these teachers’ professional subjective well-being (SWB) was affected by different factors (e.g., national policies, teacher-student relationships, etc.). In contrast, Simonton et al. (2021) applied ecosystem theory to the study of physical education teachers’ emotions and proposed a conceptual framework for understanding physical education teachers’ emotions. It is worth noting that language teachers’ emotions are more easily affected when educational situation changes greatly. Some scholars have focused on teacher emotions during curriculum reform, new policy enactments, or the recent COVID pandemic. For example, Liu et al. (2022), noting that live teaching in the context of the COVID pandemic posed new challenges to teachers, interviewed 12 Chinese high school EFL teachers and drew on Bronfenbrenner’s ecosystem theory to reveal the factors that cause teachers’ anxiety. The results indicated that teachers mainly experienced anxiety caused by technical support and health issues in the macrosystem, anxiety related to school and parents in the exosystem, and anxiety caused by teachers’ own weak modern technological skills and ineffective teacher-student interaction in live teaching in the microsystem.

There are many similar studies such as (Mercer, 2020; Sun and Yang, 2021; Li and Zhang, 2023).

The above literature shows that research on teacher emotion from an ecological perspective has been conducted in several countries in recent years. Compared with several teacher emotion models mentioned above, the ecological model emphasizes the specific situations, events, and people in which teacher emotion occurs. This helps researchers analyze the influencing factors of teacher emotion at different levels. Combined with this study’s objective, we believe it is appropriate to use an ecological perspective. However, there are still several shortcomings in the current research. First, most of the subjects are high school or university teachers, and rural elementary school teachers do not get enough attention. Second, there is a singularity of research methods and most studies adopt interviews. However, limited by interview time, the variety and quantity of data collected are not rich enough. Teaching diaries often record a stage of their work and daily trivialities. Such paper materials can serve as a data supplement and facilitate the researcher to listen to more teachers.

## 3 This study

### 3.1 Analytical framework

Ecosystem theory was proposed by Bronfenbrenner (2005), who argued that people are affected by many factors in complex environment. Ecosystem theory nests individual development within a series of interacting environmental systems. The five nested environments from near to far are Microsystems, Mesosystems, Exosystems, Macrosystems, and Chronosystems. The theory provides guidance for studying the relationship between teachers and their developmental environments. Many scholars have used this theory to analyze teacher psychology and teaching practices, such as teacher resilience (Wang and Lo, 2022), teacher retention (Zavelevsky and Lishchinsky, 2020) and so on.

**Figure 1:**
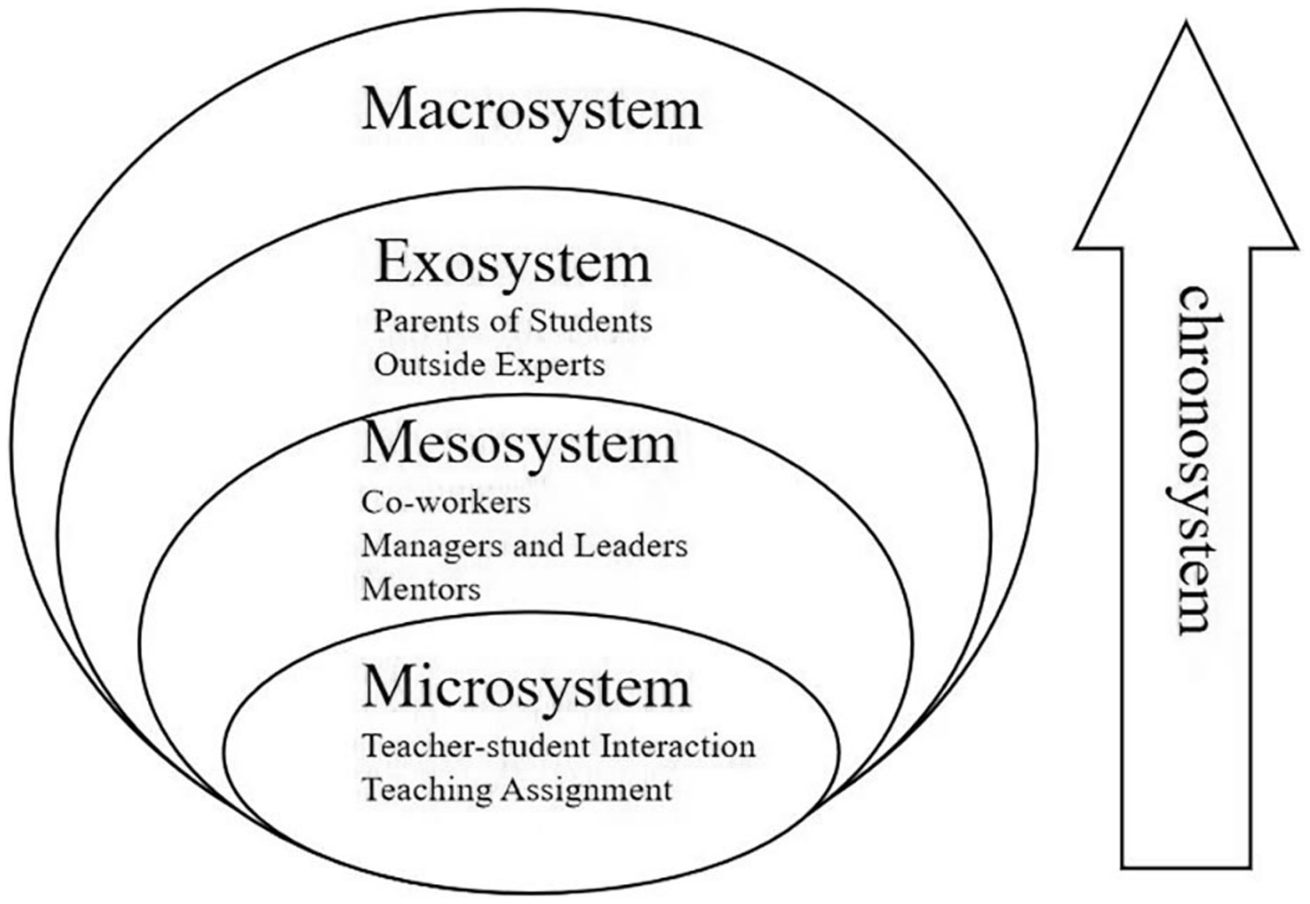
Inspired by Bronfenbrenner (2005) and Chen (2019), we propose an analytical framework to explore rural novice teachers’ emotions at the beginning of their work.

The two novice ELF teachers in this study have experienced different environments and identities during their 3 years working in rural schools. To study their emotions, we need to place them in a specific environmental identity and consider the influence of different factors. Therefore, this paper chose this framework to study the emotional development of rural novice EFL teachers and to understand the potential factors that influence rural teachers’ emotions. Inspired by Bronfenbrenner’s ecological system, we proposed an analytical framework (see Figure 1).

The interaction of rural novice EFL teachers’ emotions with each level of the system is as follows:

The microsystem is the innermost layer of the entire ecosystem. It refers to the processes that occur in the immediate environment that contains the developers (e.g., classrooms and playgrounds) (Bronfenbrenner, 2005, p. 80). The objects of this study are rural novice teachers. Their roles, classroom performance, work tasks and relationship with students are all in the Microsystem, which brings different degrees of influence on their emotions.

The Mesosystem contains connections that occur between two and more environments (Bronfenbrenner, 2005, p. 80). The system is connected to the Microsystem and includes transactions within the Microsystem. For example, interactions between teachers and school administrators and leaders can have an impact on teachers’ emotions. In addition, teachers, especially novice teachers, seek professional guidance or collaboration in larger settings, which are components of the Mesosystem (Tissington, 2008). Therefore, in this study, the connections of novice teachers in the Mesosystem with colleagues, teaching researchers, and others has become our focus.

The Exosystem includes connections of multiple environments, at least one of which does not typically contain the developing person, but in which events occur that affect processes in the immediate environment containing that person (Bronfenbrenner, 2005, p. 80). The Exosystem can be understood as an extension of Mesosystem, a broader external environment. Teachers may interact directly with factors in Exosystem or indirectly, but all of these factors trigger teacher emotions (Chen, 2020). In this study, the Exosystem includes rural teachers’ connections with students’ parents, outside experts, and rural community.

The Macrosystem is “the overall pattern of ideology and organization of social institutions shared by a particular culture or subculture” (Bronfenbrenner, 2005, p. 81). The socio-cultural background of teachers’ work and the promulgation and implementation of some education-related policies are included in the macrosystem.

The Chronosystems represents temporal change, which allows one to identify, individually or sequentially, the impact of various prior experiences on subsequent development (Bronfenbrenner, 2005, p. 83). Not only the environment in which one is placed, but also the connections between people and things can change over time, and consequently, the mindset can change. In this study, novice rural EFL teachers have different experiences at different stages. Therefore, their emotions change as time advances.

### 3.2 Research methodology

Our research question is to explore the emotional development and influencing factors of rural novice EFL teachers. Since the development of human emotions is relatively complex, it is challenging to review emotions in isolation from specific situations and events, so it is not easy to achieve our research purpose by relying on quantitative means. Narration allows teachers to recall past experiences and express emotions (Yuan and Lee, 2016). The focus of narration includes the narration of personal experience and the narration of the external environment (such as society, culture, and system). Personal experience is shaped and expressed in it (Clandinin and Rosiek, 2007, p. 42). It can help researchers understand teachers’ emotional experiences (Leigh, 2019). In addition, as one of the qualitative research methods, case studies often involve multiple contexts, allowing researchers to keep a long-term data record of the subject (Creswell and Creswell, 2017, p. 51). Unlike quantitative research methods of large-scale questionnaire distribution, the case study emphasizes the depth of analysis of research objects. In comparison, the latter emphasizes the breadth of sample size. So, the sample size of the case study is smaller, but it helps to have a deeper understanding of the “case.” As scholars have said, emotions are individual, and case studies and narratives can help capture the unique qualities of teachers’ emotions (Fried et al., 2015). We believe that the narrative case study method is suitable for this study. In addition, we also adopted the suggestion of Fried et al. (2015), which recognized that teachers’ emotions would change with the passage of events, and adopted longitudinal research.

### 3.3 Participants

The purpose of this study was to investigate the emotional development of novice EFL teachers working in rural elementary schools. We chose to identify the participants in a purposive sampling approach, i.e., typical cases that could provide rich information for the study to elucidate the research questions (Patton, 2002, p. 230).

Two novice EFL teachers, Mr. Chen, and Ms. Wu, worked in separate rural elementary schools in Zhejiang Province. Both had bachelor’s degrees and the schools were surrounded by mountains. Chen returned to his hometown after studying at a teacher training school and landing a teaching job at an elementary school in an autonomous minority region. He received top scores on the teacher recruitment exam due to his excellent undergraduate training. Over half of the school’s students are from ethnic minority backgrounds. Wu applied to be a city elementary school teacher but did not get in due to lower scores. She now teaches at a township school serving students from different villages. The township’s elementary school is far from the county town, with a three-hour bus ride. It’s further out than Chen. The school is a boarding school, with students only going home on weekends and holidays. Chen and Wu work as teachers in a small rural elementary school with basic modern teaching tools. The school reduced the number of first- and second-grade classes from six to three per year in Chen’s second year of teaching. Over 70 teachers work at the school. Wu’s school only have 37 teachers, and 20 students per class. Teachers live in the school’s dormitory. Most students live with older relatives due to parents working outside.

Two novice EFL teachers willingly participated in the study, cooperated with us, and shared their emotional experiences in the rural school. They provided insights into their three-year tenure at a rural elementary school. The researcher was familiar with the participants, which helped facilitate the study and provided a deeper understanding of their experiences working in rural areas. Based on these factors, we determined that these individuals were appropriate research subjects.

### 3.4 Data collection

Before the research started, we informed the participants of the purpose and significance of the study by telephone, assuring them that all data was for research purposes only. All participants signed informed consent forms. The data collected within the 3 years came from a wide range of sources. Our researcher collected data through semi-structured interviews, narrative frameworks, and teachers’ reflective journals. In addition, the researcher maintained close contact with the participants through wechat and email during the duration of the research. Informal conversations conducted on social software and the researcher’s memo notes supplemented the data. Although not systematically analyzed, these data could be used to clarify ambiguous expressions in interviews and diaries, as well as to provide insights for later data analysis.

#### 3.4.1 Semi-structured interviews

Personal thoughts cannot be directly observed, but through interview, it helps the researchers to understand the inner thoughts of the interviewees (Miles et al., 2014). Semi-structured interviews are more common in case studies and have been used to explore teacher perceptions and teacher emotions (e.g., Li and Craig, 2019; Gao and Zhang, 2020). Semi-structured interviews are the main data source of this study. Because our participants are working in rural areas, we conducted one-on-one online interviews with the participants, taking into account the distance and other factors, and all interviews were conducted in Mandarin Chinese. The interviews were conducted using Tencent conferencing software, which is commonly used within China. During the 3 years of the novice EFL teacher’s employment, we conducted a total of six interviews, one at the end of each semester, and each interview lasted about 2.5 h. In the last interview, we added a session, which was to review and summarize the emotional changes during the 3 years with the participants. Before each interview began, the interviewer would reconfirm the purpose of the study, indicating that participants could withdraw at any stage during the interview if they felt uncomfortable. The content of each interview can be roughly divided into three parts. In the first part, we used the reflective journals provided by the participant as clues and asked the participant to describe the emotional experiences of being a novice teacher during the semester in chronological order. Based on their description, the researchers further pursued the situations, details, and feelings at that time. The second part focused on interviewing the participants about the memorable events they experienced. The interviewer guided participants to recall significant events they encountered in their work and the emotions that were triggered by those events. In the third part, participants freely described their experiences with different people around them (students in the Microsystem, colleagues, administrators, parents of students in the Mesosystem, etc.). The interviewer asked some open and exploratory questions at the right time while listening. With the permission of the participants, all online interviews were video-recorded and transcribed in text, and we ended up with an interview material of approximately 150,000 words.

#### 3.4.2 Narrative framework

Researches on EFL teachers’ emotions suggest that narrative is a tool for researchers to capture teachers’ thoughts and understand their behaviors (Wolff and Costa, 2017). Narratives can help people understand how past experiences and emotional experiences influence teachers’ current behaviors and perceptions (e.g., Barkhuizen and Consoli, 2021).

The narrative framework of this research design is as follows (see Table 1).

**Table 1:** The narrative framework of this study.

Stage	Contents
After each semester	The most positive emotional experience The most negative emotional experience Specific events that trigger emotional experience The key person who triggers the emotional experience The impact of this emotional experience

#### 3.4.3 Teachers’ reflective journals

All participants in the study provided us with reflective journals, totaling over 50,000 words. The reflective journals recorded their work experiences in three different stage as novice teachers in rural areas. The researchers focused on the content of emotion in the reflective journals, and further extracted and recorded it to understand their real emotional experience in rural teaching.

### 3.5 Data analysis

We used three-step coding (Corbin and Strauss, 2015, pp. 215–310) and thematic analysis (Braun and Clarke, 2006) to analyze the collected data. Nvivo12plus was the main tool used to code the data in this study. Firstly, the researcher integrated the transcribed interviews and the extracted emotional diaries in a single document. All sentences and keywords about emotions and emotion-generating contexts (events that interacted with the environment) were coded by reading several times. We also created a code book to record and correspond the teacher’s emotions with the time, context, event and person of the emergence of emotions by secondary recording. This identified the relationship between different emotions and different levels of the environment, and 289 codes were generated in this session. After discussion, we made some modifications. Secondly, we referred to some scholars about the classification of emotions, kept close to the research question, and classified all nodes into 62 emotions. Finally, guided by the Bronfenbrenner ecological framework, we divided the interactions between participants and environments into four systems (Mcrosystem, Mesosystem, Exosystem, and Macrosystem). The coding phase was performed by two researchers (authors) and three educational graduate assistants. Each researcher coded individually. After all the coding work was completed, all of researchers attended meetings to discuss, negotiate and modify when there are disagreements, until everyone’s opinion is unified. To ensure the credibility of the data, we used data triangulation techniques. In addition, we sent the extracted sentiments and themes to the participants in a timely manner to ensure that there were no ambiguities throughout the process, that our interpretation of the data was consistent with what they really mean, and to obtain their approval of the data interpretation (Table 2).

**Table 2:** A sample of data coding steps.

Research questions	Original material	Emotion	Opening coding	Axial coding	Selective coding
1. What kind of emotional experience did novice EFL teachers have during the first period of working in a rural primary school?	Due to my lack of experience, I felt nervous when I was teaching, and I felt my palms sweating in the face of the students’ attentive gaze. (Chen-The early interview)	Nervous	Teaching stress caused by inexperience	Teaching Assignment	Microsystem
2. What ecological factors may affect the emotional development of novice teacher teachers in rural areas?	The experts offer a lot of advice and experience in teaching, which is exactly what a novice teacher like me needs. In the interaction with experts, I feel motivated and look forward to becoming an excellent teacher in the future (Wu-The third interview)	Inspired	The experts gave encouragement and constructive advice	Expert support	Exosystem

The highlighted part is the teacher’s emotion, and the underlined part is the triggering event of the teacher’s emotion.

## 4 Results

During the 3 years of two novice EFL teachers Chen and Wu’s teaching practices in rural primary schools, the narrative analysis of the interview data reveals that both of them have tackled emotional experiences: 62 emotions are identified, including 35 positive ones and 27 negative ones. In addition, according to the statements of two novice teachers, Chen appeared to be more positive than Wu Interpretive report is given as follows.

### 4.1 Microsystem

According to previous studies by other scholars, the Microsystem environment has the greatest influence on teachers’ daily actions, in which they could approach the maximum amount of emotions. The two novice EFL teachers (in this study) have contacted with students most frequently during their 3 years of teaching in rural areas. During their interactions with students, 19 positive emotions (happy, excited, expectant, relived, pleasant, proud, curious, surprised, satisfied, moved, enjoyable, intimate, loving, confident, motivated, hopeful, fulfilled, love, peaceful) and 9 kinds of negative emotions (angry, anxious, nervous, self-condemned, exhausted, frustrated, regretful, confused, dissatisfied) are identified.

### 4.2 Teacher-student interaction

One of the strongest feelings Chen and Wu mentioned several times during the interview is that interacting with students brings them happiness and satisfaction. “Students make me think that teaching is happiness.” Chen said. Chen added that the sense of happiness and satisfaction actually came from some everyday and even trivial things, but mostly came from students’ good performances. Students answer questions voluntarily and ask confused ones actively after class. When it comes to exercises or homework, I also see their carefully written notes or modifications, and the classroom is always neat and clean. These moments are the ‘placebo’ of ordinary life and make me feel happy.” When asked about satisfaction, Chen replied, “I get some feedback from their daily performance, such as books with densely written notes and neatly organized test papers. This attitude toward learning and thirst for knowledge fulfills one of my original ideals as a teacher -- to make students happy to learn. And of course it keeps me motivated.” Wu also wrote in her diary that she was not only a teacher, but also a mother, a listener and a role model. Some students’ parents are too bound up in their work to company their children and only go home during the Spring Festival, which has caused some adverse consequences in students’ character formation. “Knowing how much they lack parental care and concern deep inside, I hug and encourage them whenever they need it. By doing so, I also feel the warmth of interaction from my students.” Chen also mentioned that when he first took over his new class, he felt a little nervous about meeting unfamiliar faces. In the first class, he introduced himself by video, secretly observing the students’ reactions as he played the video. It was clear that the students were also curious about the young teacher, and he was a little excited. In the later days, students and Chen became familiar with each other from strangers. “Because I am a young teacher, I have more common topics with my students. I can joke and chat with them occasionally, and gradually I have a sense of intimacy” (Chen-interview).

The two teachers also had negative emotions. Chen recorded a negative emotional incident in his diary. One afternoon, he took the students to the playground to do some free activities since all teaching tasks had been finished but there’s still time left. But one of them fell down and hurt his ankle while playing. Chen’s first reaction was nervousness and self-blamed. He was worried about the students’ injuries and that he would be blamed by the student’s parents or the school. Because it was he who took the students to the playground without permission that led to the accident, he fell into deep remorse and regret. As a novice teacher who lacks empirical emergency reaction, Chen said he felt at a loss at that time. So he turned to experienced teachers and took actions immediately following the suggestion and instruction offered by them. He sent the student to the hospital in time, reported the matter to the class teacher, the principal and the student’s parents, and went to check the monitoring to find out the reason. Later, when he saw the student returned to school healthily and greeted him with a smile, Chen felt relieved.

The most common negative emotions described by both teachers were anger and frustration. “As a new teacher, I think my lack of experience sometimes makes me unable to handle problems well. The few who are particularly naughty give me a headache. I try to communicate with them when they do not pay attention or do not finish their homework on time, but it does not seem to work. They just keep going and it annoys me” (Wu-Interview). Both novice teachers give their animadversions and guidance on students’ misbehavior. Wu often worries about whether her criticism will affect her relationship with her students. Chen said, although he criticized harshly, but he was soft in his heart. While, Chen and Wu’s frustration mainly comes more from the students’ feedback on their grades. Chen expressed his frustration with the disparity between his efforts and his rewards. “I feel like I’m already trying so hard to teach that my throat is often hoarse. But my students are not doing well or living up to my expectations, which have somewhat dampened my enthusiasm and original intention of teaching” (Chen-interview).

### 4.3 Teaching assignment

Teaching tasks are an important part of a teacher’s job. In the first 3 years of work, the two teachers were closely involved in and completed a number of teaching tasks, and their emotions were also affected by the teaching tasks.

We find the mental activities of the two teachers’ first class in their diaries. “I gave my first class today. Although there have been mock classes in college, it did not feel like this at all. I felt students’ eyes on me and my palms sweating.” “If you do not have a plan for what students are going to ask you in class, you do not know what to do. My first year at school, I used to take a deep breath before every class and it’s much better now…. There are also open classes where the school leaders and senior teachers sit in the back row and I almost ‘hold my breath’ while listening to students’ questions because I’m afraid I’m not getting the point across” (Chen-interview). As a novice teacher, the open class is a teaching task that must be completed. Both teachers mentioned that they felt overloaded when preparing for the open class. Wu also told us about a brief lapse in her public class due to nervousness -- “I forgot my words! I was talking and suddenly I did not know what the next session was. The more nervous I felt, the less I could recall. I still remember how awkward it was when I froze at the podium for several minutes.” When the teaching did not work out, the two teachers both expressed their confusion and upsetting emotions.

Of course, positive emotions were also mentioned by the novice. “If I give a good lesson, I feel satisfied! I feel especially happy when getting positive feedback from my students. They show a lot of interest in my instructional design, and teaching steps proceed smoothly without any obstacles” (Chen-interview). When the teaching process is smooth and the students respond positively to the class, two new EFL teachers will feel that their teaching is half done. In the interview, they believed that the main source of their positive feelings was the effectiveness of teacher-student interaction in classroom teaching. Chen said, “Although it may sound vague, it is clear to me that a successful class is one in which the students’ thinking is moving with the teacher, rather than just following along. Effective teacher-student interaction is especially important for novice teachers like me, and it’s an indicator of my reflection after class. I feel a sense of accomplishment when I interact effectively and fully with my students and when the class is working well.” Teachers’ positive emotions also depend on how much control they have over the teaching. Wu shared her practice in the statement, “In the first place, I was unable to get control of the class for the unfamiliarity with material content and teaching methods. After 3 years of training, I am fully familiar with the textbooks and learning situation, and I have become more confident and calm in teaching.”

### 4.4 Mesosystems

#### 4.4.1 Co-workers

In addition to daily teaching and contact with students, teachers also have close communication with colleagues. During the 3 years of working in a rural primary school, two new teachers have gained friendship with colleagues, most feelings appear to be positive, though negative ones are also indispensable.

“There are young teachers and old teachers in the office… In fact, There are not many English teachers in our school, so we do not have to worry about working competitions or point-scoring in the workplace. Teachers are getting along very well. However, it also brings the problem that it is difficult find English teachers of the same age to communicate with each other, which makes me feel a little dull and boring. When I see English teachers who are better than me, I feel stressed and anxious, and even self-deprecating” (Wu-interview).

In addition to Wu’s experience, Chen also shared his associations with colleagues. Inclusive workplace and feeling relaxed were emphasized. What was repeatedly emphasized was that he felt relaxed getting along with his colleagues. “I appreciate that Ms. Huang and Ms. Zhang are willing to spend their rest time to help me make teaching aids before for the open class. I feel so happy to be in such a united group, and I certainly will not hesitate to help my colleagues when they need help” (Excerpt from teaching diary). In the first year of working, Chen occasionally received criticism or advice from senior teachers. Choosing not to retort or argue for his opinions, Chen acted calmly and listened to them with patience. Later, when Chen’s teaching was generally recognized and praised by his colleagues, he was happy.

#### 4.4.2 Managers and leaders

In addition to colleagues, teachers interact with managers and leaders. Chen and Wu describe their mixed feelings about interacting with managers and leaders. On the one hand, Chen said that since he joined the school, the administrators and leaders of the school have been very caring and have given a lot of support to the novice teachers, both in terms of teaching work and life, which moved him a lot. In particular, when the designed class for competition won a prize in the city, Chen was recognized by the leaders and rewarded by the school, which made him feel proud and proud. Wu also mentioned the joy he felt when leaders praised him. On the other hand, two novice teachers expressed their dissatisfaction and incomprehension. The school seems to regard novice teachers as a group that preserves energy and awaits exercises. Since we do not need to go home to look after the children yet, they dump everything on us. There was a time when I was preparing for an open class competition downtown, and the director of research gave me 3 days to write a teaching research paper. He did not consult me in advance or sent any other teacher for assistance, which caused my internal struggle and a feeling of resistance. I just wanted to finish the open class competition but I obeyed him anyway. I felt helpless in those days, because I had not systematically studied research paper writing before.” Teachers often have no choice but to accept the tasks assigned by their superiors. But two new teachers say they are happy to accept tasks that are within their abilities and feel a sense of accomplishment when they complete them. However, they tend to have negative emotions such as boredom, resistance, confusion and even dissatisfaction when they are urgently assigned tasks that are beyond their ability or meaningless. “Maybe out of a utilitarian idea, when my superiors assign me to do some tasks that are not helpful to my promotion, I recognize them as meaningless, and I will not spare that much of efforts when completing these tasks” (Wu-interview).

#### 4.4.3 Mentors

Novice teachers are assigned mentors during their initial period of employment. Mentors are usually experienced veteran teachers with excellent teaching achievement who guide the novice teachers in their teaching and work. The two novice teachers produced X kinds of emotions during their interactions with their mentors. Wu felt uneasy when she first met her mentor. “I thought he was a serious person because we were strangers when we first met, but I was flattered by his gentleness and patience” (Wu-Inerview). With the guidance and help of his mentor, Wu is more unhurried and determined.

Among these emotions, admiration was the common emotion repeatedly emphasized by the two novice teachers. They admired their mentors’ superior teaching skills and ability to handle matters. “My mentor is the head teacher in charge of the whole grade group. I have listened to his classes and the teaching design is very clever. The English scores of students in his class often rank first in the grade, and he has won many awards, which I admire and envy” (Wu-interview). “My mentor helped me a lot in teaching, and she was always the first one to come forward and give me advice when I had a problem. During the preparation of the open class, she rehearsed with me repeatedly, and I could strongly feel that she wanted to make my teaching better. I am very grateful that we often communicate with each other about teaching, and I am greatly encouraged by her suggestions and affirmations. I would not have progressed as fast without her” (Chen-interview).

### 4.5 Exosystem system

#### 4.5.1 Parents of students

Two novice teachers explained to us that most of their students’ parents work outside the home, and the students usually live with their grandmothers or other relatives, so they are often in contact with their students’ parents by phone or WeChat. In interviews, two novice teachers mentioned to us the complex emotions of interacting with their students’ parents. “I am not satisfied with most of my students’ parents. It seems to me that they focus on their work rather than on their children’s education.” The parents’ lackadaisical attitude toward their children’s English learning caused some frustration for two novice EFL teachers. “Once I called a student’s father to report to him about his child’s recent poor performance in school and his failing English grades. As a result, he said ‘I do not think poor English grades will have any impact on his future, so if he really cannot learn it well, I do not want to force anything.’ I ended the call calmly, even though I was on the verge of losing control of my anger” (Wu-interview).

But not all parents are like this; there are some parents who make teachers feel different emotions, and Chen described to us one parent of a student who made a profound impact on him. “The grandmother of a student in our class impressed me deeply. This elderly person, who has no education and cannot even speaks Mandarin fluently, takes her granddaughter’s studies very seriously. After every test, she is always the first parent to call me to ask about her granddaughter’s grades and studies. Once the old person met me when she picked up her granddaughter from school, and she said thanks for my work. Although she did not know what English is, she still pleaded me to give more guidance to her granddaughter’s English study, which was as important as other subjects. I could feel her respect and trust in me, and such a parent is very cooperative with my work.” The parent’s respect and trust in the novice teacher and their unbiased view of English as a subject gave Chen a surge of relief.

In addition, the two novice teachers agreed that they were not good at interacting with parents when they first started working. “Maybe novice teachers are a little timid because the parents are a little older than us, so you know. At the beginning I was cautious in communicating with parents, fearing that I would say something wrong. But after more contact, that tension eased and I was natural and spontaneous in my interactions with parents” (Chen-interview).

#### 4.5.2 Outside experts

In China, each administrative district has a special educational administration and teaching-research experts. Teaching-research experts are a distinctive group of Chinese teachers. In addition to their high-level teaching skills, their theoretical research ability is more solid than that of primary school teacher. Meanwhile, they participate in the district’s teaching quality testing and curriculum development. Especially since the implementation of the new curriculum reform in China, the role of teaching-research experts has become particularly important, as they play the role of refiners and facilitators of regional education and teaching. In the past 3 years, the two young EFL teachers have participated in many urban teaching and research activities (e.g., expert lectures, teaching materials workshops, etc.), and the teaching-research experts have also investigated their rural elementary school. Participants described to us the positive emotions arising from contact with outside experts: rewarding, hopeful, longing, adoring, eager, and excited.

“I was nervous and excited to observe a provincial teaching materials workshop. I saw many qualified and excellent fellow teachers. I took notes carefully while listening to the lectures. I benefited a lot from the experts’ interpretation of teaching materials and analysis of curriculum standards. I would have a feeling of ‘clearing the fog’, that is, being inspired to solve the confusion I had encountered in my teaching in the past” (Chen-interview). Another teacher, Wu, expressed a positive attitude toward participating in such teaching-research activities. “Although I am only a novice teacher, I am eager for a bigger ‘stage’. I feel very excited and motivated after attending the lectures by experts. I hope I can become a teaching expert in the future, I am looking forward to it” (Wu-interview). We also see similar thoughts in Chen’s diary. “I have an urge, a passion, to incorporate their ‘golden ideas’ into my own daily teaching, and I want to be like them.”

#### 4.5.3 Macrosystem

The data shows that the two teachers produced the least amount of emotions at this level. The two novice EFL teachers experienced a total of three positive emotions (love, relaxation, happiness) and three negative emotions (challenge, difficulty, boredom). They said the working atmosphere in rural primary schools was relatively relaxed. Chen confessed, “My college roommate went to work in a famous primary school in the city after graduation, and we chat occasionally. He always has a busy schedule, compared to my job, which is easier and I have more time to myself. The days working in a village primary school are ordinary, but I felt a burst of happiness from time to time, especially when I stay with my students.” Wu mentioned that the pressure of not being as competitive for careers as in urban primary schools is one of the reasons he stays in rural schools. “And the countryside is not as crowded and noisy as the city, so I do not have to get up early and squeeze into the subway. And the people I met are honest and kind. I even went to a student’s grandmother’s house for dinner” (Wu-interview).

However, it is precisely because of working in a rural primary school that Wu has produced some negative emotions. “It’s been a while since I’ve been here, and there are still a lot of things I do not get used to. It’s hard to communicate with the local parents, most of whom speak the local dialect, so it seems I have to learn the dialect here…. Weekends are boring, there is no gym and no cinema” (from Wu’s teaching diary). In addition, social and policy factors also have an impact on the emotions of the two teachers. Society and policies pay more and more attention to information-based teaching, and the insufficient support for information-based teaching in rural primary schools has caused negative emotions for the two novice teachers. “Now society is more concerned about information-based teaching than before, especially after the pandemic. In recent years, China has continuously introduced education policies emphasizing the integration of teaching and information technology. This poses certain challenges and difficulties for me. Our school does not have enough modern teaching equipment, and many urban schools have what our school does not have. And I think the principal does not pay enough attention to the development of teachers’ digital literacy”.

In short, the emotions of two novice EFL teachers can be influenced by the environmental climate, policy system, etc.

#### 4.5.4 Chronosystems

During the 3 years of working in rural primary school, two teachers experienced changes in their inner emotions as they came into contact with different people and things at different stages. Our teachers’ emotional changes are divided into three stages (the first year, the second year, and the third year of working in rural areas).

In the first stage of their work, the two novice EFL teachers were very new to the school and the students, and were nervous and excited to be out of the “ivory tower” and into the formal classroom. Chen described himself as an “observer and imitator,” always observing experienced teachers and his own students. He applied the teaching techniques he observed in the class of other excellent peers to his own teaching practice, and constantly explored and summarized in imitation. By observing students, he tried to understand different students’ personalities, which helps to bring teachers and students closer to each other. Chen has an outgoing personality and quickly got familiar with people around him. In the process of interacting with students, colleagues and tutors, he had gained a lot of positive emotional experience. However, he felt frustrated and even depressed when his students’ test results were not satisfactory or his teaching did not achieve the expected results. Being given multiple tasks to complete in a short period of time also left him feeling overwhelmed.

In the second stage of their work, the two novice EFL teachers gradually became familiar with their surroundings and gained a basic understanding of their students, colleagues and the school. They began to participate in some open class competitions and teaching-research activities frequently, which brought some pressure to them. But they were exposed to more excellent peers in these competitions and activities, and were inspired to reflect more on their own teaching. Both novice teachers had made new efforts. Wu realized that part of her negative feelings came from a lack of achievement in teaching, and she began to make some teaching plans, such as finding ways to get students to speak English and delving into English reading skills. Chen helped students learn efficiently by retelling the framework of the text in comic strips, organizing grammar points with mind maps and other ways. “More and more students are raising their hands in class and becoming interested in English” (Chen-interview). The initial results of the teaching and the enthusiastic help of their mentors made the two novice teachers very encouraged.

In the third stage of their work, the two novice EFL teachers have been able to handle various internal and external matters independently, and became more and more proficient in their daily teaching. Moreover, Chen won a prize in a teaching competition, which gave him a strong sense of accomplishment for seeking external recognition. The two novice teachers have found the joy of work and life in rural schools, and getting along with the simple and kind rural students makes them feel happy.

Wu sees herself as a “transformer” who is no longer self-doubting and who has made progress from her 2 years of training. She sums up her emotions in the third stage of her work with “confident and relaxed” and “happy.” Chen believes that he is no longer a simple “imitator” role, but has become a “reflector.” He is refining his own teaching style based on learning from others’ teaching thinking and value orientation, combined with his own reality. And he is looking forward to the future. In conclusion, both novice teachers believe they have made progress in these 3 years, but there is still much room for improvement.

## 5 Discussion

The two novice EFL teachers in the study had frequent and complex interactions with students, school leaders, parents, colleagues, Etc. during the 3 years they worked in rural primary schools and described a rich and diverse emotional experience to us. Combined with our findings, it is not difficult to understand that some scholars consider teachers to be professionals of “high emotional labor” (Kang, 2020). In this study, the two beginners EFL mentioned the most emotions in the microsystem, followed by the intermediate, external, and macro systems. The emotions mentioned in the time system accompany the novice teacher’s work process. In describing the negative emotions, two novice EFL teachers spoke about the multiple challenges they encountered in their work. For example, some rural children show higher emotional needs than urban children of the same age, which requires teachers to play multiple roles, such as surrogate parents, listeners, and psychological counseling rooms, bringing role pressure to them. And the lack of parental support and effective home-school communication.

Furthermore, novice teachers rarely mobilize the resources around them to deal with the difficulties they encounter in their work (Mansfield et al., 2014). So, in their first year on the job, the two novice teachers were constantly troubled by negative emotions such as tension and anxiety. This seems to support previous research suggesting that novice teachers experience high anxiety and stress early in their careers (Mevarech and Maskit, 2015).

It can be seen from the results that daily interactions with students generate the most emotions (e.g., anticipation, happiness, anger, etc.) for teachers, which is consistent with previous studies (Chen, 2020). One of the essential factors affecting teachers’ emotions is student performance. Student achievement is a thin thread that affects teachers’ emotions, especially in China. Both new teachers in the study said they wanted to prove their teaching ability through student achievement. When students’ grades do not meet their expectations, they may experience negative feelings such as depression or self-doubt. When students behave poorly (e.g., disobey instructions and have behavioral problems), they feel angry. Previous studies have pointed out that students’ misbehavior is likely to bring negative emotional experiences (such as stress and emotional exhaustion) to teachers (Tsouloupas et al., 2010). In the study of Sutton and Wheatley (2003), teachers’ positive emotions are closely related to students’ good performance, and teachers will feel happy when students act proactively and responsibly. When students experience success, both teachers and students feel happy. Clark et al. (2003) describe this emotion as “empathic.”

It is worth mentioning that too many administrative matters and urgent task schedules may cause dissatisfaction among novice teachers. Both novice teachers in this study had been assigned to complete certain tasks. When given tasks unprepared or in a time crunch, they experience high levels of stress, anxiety, or helplessness. If the task itself was not related to their improvement in teaching ability, they became bored and dissatisfied. Such negative emotions affect their ability to complete tasks. They tend to perform tasks that they consider unimportant and worthless in a casual manner. The two novice teachers urgently hope that their superiors can give them more time to focus on teaching work, rather than dealing with trivial affairs that consume personal time and energy. After all, too many administrative tasks can squeeze out the time teachers spend on teaching (Kim, 2019).

## 6 Conclusion and implications

This study makes a longitudinal and holistic exploration of the emotions of novice EFL teachers in rural primary schools. From the ecology perspective, this paper introduces the interaction between novice teachers and different environments and the resulting emotions. In this study, we learned that the emotions of novice teachers in the initial stage of work are strong, complex, dynamic, and multi-dimensional. This is the same as previous research (Anttila et al., 2016). According to the research, in the 3 years of working in the rural primary school, the two novice teachers had a total of 35 positive emotions and 27 negative emotions, and all kinds of emotions interwoven through their teaching work. Despite the negative emotions, the two novice EFL teachers self-regulate, and the success they experience in teaching gives them internal motivation. Therefore, from a vertical perspective, the mood of the two teachers changed from negative to positive, from anxiety and tension in the early stage of work to peace and calm in the later stage. Looking at the entire ecosystem, teachers developed the most emotions in the microsystem. Focusing on our other research question, the factors affecting the emotions of two novice EFL teachers can be divided into internal and external aspects. Internal factors are the sum of characteristics related to teachers, including teachers’ educational beliefs, self-expectations, personality characteristics, and so on. The teacher’s emotional regulation ability has a more significant influence than all the internal factors. According to the classification of Taxer and Gross (2018), the emotion regulation strategies of the two novice teachers in this study mainly include situation correction and attention allocation. For example, Wu changed the unsatisfactory teaching effect by asking for help from tutors and trying new teaching methods. In addition, the two teachers focused on their growth and devoted themselves to teaching, which lessened the boredom of working in a rural area and increased their enthusiasm. External factors include student performance, school climate, external expectations, parental support, and educational policies. A harmonious school atmosphere (such as mutual help among colleagues and care and support for novice teachers) can promote teachers’ positive emotions, and parents’ attitudes toward teachers and cooperation in action will also affect teachers’ emotions.

This study elaborates the story of two novice EFL teachers’ experiences of working in a rural elementary school for 3 years. Through qualitative analysis, it investigates their emotional experiences and influencing factors arising from their interactions with different ecosystems in the early stage of their work. This study has the following implications for future research and teacher education.

First of all, teachers’ emotional regulation ability is very important. It is necessary to improve primary school teachers’ understanding of teaching emotion. Activities related to teachers’ emotional regulation can be incorporated into pre-service teacher training. For example, teaching teachers strategies to regulate emotions by offering relevant lectures will help to promote the emotional health and professional development of primary school teachers.

Secondly, novice teachers in this study mentioned that too much non-teaching work squeezes their time, and the non-teaching burden of teachers deserves our attention. Previous studies have indicated that there is no direct relationship between teacher workload and school education outcomes, but there is an indirect relationship with job satisfaction, organizational commitment and professionalism. That is, teachers’ teaching satisfaction, classroom involvement and professionalism will increase with the reduction of administrative workload (Lee and Lee, 2010). In other words, increased administrative workload will weaken teachers’ focus on teaching activities, leading to deprofessionalization (Kim, 2019). Therefore, reducing teachers’ unnecessary or formal non-teaching tasks and keeping their administrative workload within a reasonable range are of practical importance for both teachers and school development.

Research on the professional development of novice teachers, especially those working in remote and less developed areas, needs to be further deepened. Two novice EFL teachers in this study admitted that they had difficulty mobilizing resources around them to improve themselves because of limited regional resources. In the rural context, teachers usually use vacation time to go to urban schools for further study, which is undeniably reasonable but it has some problems (such as round-trip distance, time cost, financial support, etc.). Some scholars have proposed the characteristics of effective professional development of teachers, one of which is that professional development should have sufficient duration (intensity and contact time) (Wilson, 2013, p. 310). Obviously, it is difficult to keep teachers in a state of continuous learning in the way mentioned above. Maher and Prescott (2017) discussed the application of video conferencing to the professional development of rural teachers. Through qualitative data analysis, it reveals the advantages of VC applied to the professional development of teachers in rural and remote schools (helping to reduce time and cost, providing more communication opportunities for teachers through real-time dialogue, minimizing social presence, and alleviating the sense of isolation of rural teachers). Therefore, in the future, it will be interesting and meaningful to continue in-depth research on digital empowerment of teachers’ professional development in remote areas at both practical and theoretical levels. For example, building a digital teacher professional quality improvement platform for rural novice teachers will help teachers to carry out close communication and cooperation across individuals, departments and regions. So that the novice teachers at the initial stage of their careers have more opportunities to come into contact with scholars and peers from other universities and institutions, and constantly broaden their own development space.

## Data availability statement

The original contributions presented in the study are included in the article/supplementary material, further inquiries can be directed to the corresponding author.

## Ethics statement

The studies involving humans were approved by Jing Hengyi School of Education, Hangzhou Normal University. The studies were conducted in accordance with the local legislation and institutional requirements. The participants provided their written informed consent to participate in this study. Written informed consent was obtained from the individual(s) for the publication of any potentially identifiable images or data included in this article.

## Author contributions

YY: Conceptualization, Data curation, Investigation, Methodology, Software, Visualization, Writing – original draft. JX: Investigation, Project administration, Software, Supervision, Visualization, Writing – review & editing, Validation. CY: Funding acquisition, Project administration, Resources, Supervision, Writing – review & editing.
